# A comparative numerical evaluation of linear anode arrangements for enhancing above ground storage tank cathodic protection via mesh grid and concentric ring designs

**DOI:** 10.1038/s41598-023-44759-3

**Published:** 2023-11-10

**Authors:** Mohammad Javad Shirshahi, Seyed Farshid Chini, Peyman Taheri, Abraham Mansouri

**Affiliations:** 1 School of Mechanical Engineering, College of Engineering, University of Tehran, Tehran, Iran; 2 Department of Mechanical Engineering, Higher College of Technology, DBM, Dubai, UAE; 3 Matergenics Inc, 100 Business Center Drive, Pittsburgh, PA 15205 USA

**Keywords:** Mechanical engineering, Corrosion

## Abstract

Cathodic protection as a complementary method is one of the most effective ways to prevent corrosion, along with coating and choosing the suitable material. There are different ways to protect the storage tank bottom. Due to the presence of the geo membrane layer and its effective and pivotal role, the use of mixed metal oxide (MMO) anodes is highly recommended. In the current study, the protection process of the above-ground storage tank bottom is simulated using MMO backfilled wire (concentric rings) and ribbon anodes (mesh grid system). In this regard, several parameters must be considered in reaching the protection criteria. The simulation results show that the effect of the limiting oxygen current density, soil resistivity and the anodic current have the greatest role. Anode depth and the spacing between the anodes are also critical factors if they have large values. Due to the importance of the number of conductor bars and feeding cables, two configurations have been presented for ribbon anodes. These configurations have a direct impact on grid resistance, rectifier size, and related costs. Finally, an economic comparison has been made regarding the use of sacrificial and MMO anodes in the form of ribbon next to wire anodes. The results show that, in the use of ribbon anodes, MMO anodes have better performance than magnesium and zinc sacrificial anodes, and the cost required for MMO linear anodes is far lower than the method of sacrificial ribbon anodes. In the present study, the cost of the MMO linear anode system is approximately 20% of zinc and magnesium sacrificial ribbon anode system cost.

## Introduction

The importance of using the cathodic protection method as a complementary and cost-effective approach for protection is known in the industry^1^ and the protection of the above-ground tanks bottom as storage resources is of particular importance. In the past, various methods have been proposed to protect the tank bottom, e.g. deep well anodes and peripheral distribution anodes^2^. With the advancement of materials, today the use of mixed metal oxide (MMO) linear anodes is on the agenda^3^ and next to that the role of the geo membrane layer as a protective layer is undeniable^4^.

The presence and implementation of the geo membrane layer play an effective role in distributing the current at the bottom of the tank and preventing current leakage to nearby structures where linear anodes are used. The important point is that the presence of the geo membrane layer prevents the current from reaching the bottom of the tank by conventional methods (deep well anodes and distributed anodes buried around the tank^4,5^). Therefore, the use of linear anodes along with the geo membrane layer improves the protection performance of the structure's bottom to the highest level. MMO linear anodes include ribbon and wire anodes. Each MMO anode consists of two parts: the substrate and the electro-catalyst coating. MMO anodes are applied over a commercially pure titanium substrate. The coating consists of an electro-catalytic conductive component that catalyzes the reaction to generate current flow and bulk oxides (cheaper fill materials) that prevent corrosion of the substrate material and the use of iridium oxide (IrO_2_) is preferred for all cathodic protection applications.

Anodes of this kind are dimensionally stable, have a long life, and are not consumed during the process of generating cathodic protection currents^3^. It is worth noting that ribbon anodes utilized in mesh grid systems have a rectangular cross-section and wire anodes used in concentric rings have a circular cross-section. Each of these two anodes has specific advantages that will be explained further. For a MMO ribbon, it is vital to install conductor bars to achieve electrical continuity without excessive attenuation. Also, the important role of using backfill in wire anodes and reducing the oxygen content strongly affects the performance of the protection system^4,5^. In the next section, we will examine the literature concerning storage tank bottom corrosion.

Peratta et al.^6^ with the help of Beasy Ltd. software and ribbon mesh grid, simulated the protection process of the storage tank bottom. They unveiled four designs with different numbers of supply cables and conductor bars with the same distance between anodes. The simulation results show that by increasing the conductor bars and supply cable, the voltage distribution in the anode grid becomes better and more uniform, and also the potential distribution in the tank bottom becomes more negative (about 20–30 mV) and improves. They used a shift criterion of 100 mV. They also investigated the effect of increasing the transformer rectifier voltage. They also studied the effect of a poor weld by applying or introducing a 0.5 $\Omega$ resistance in a part of the tank bottom and the negative effect of this work.

Adey et al.^7^ performed the process of simulating tank bottom protection for a ribbon mesh network using Beasy software. In this article, different spacing between anodes and different burial depths of anodes to the bottom of the tank are studied. The results show that by increasing the burial depth, the range of potential changes decreases (potential distribution becomes more uniform), and the potential of the tank bottom becomes more positive. By reducing the distance between the anodes, the range of potential changes increases, while the potential of the tank bottom becomes more negative, but at the same time, the cost also increases. As the depth of burial increases, the voltage of the anode grid increases very little (between 5 and 15 mV) on average. By reducing the spacing between the anodes, the voltage of the anode grid is also reduced to a very low value (2–5 mV).

Baynham et al.^8^ studied the external cathodic protection of oil and gas tank bottom by using the concentric circular ribbon system with rectangular section and Beasy software. To reach the protection criterion, they used the reduction of the distance between the anodes and the increase of the transformer rectifier voltage and investigated the effect of reducing the soil resistivity, and also stated how the voltage in the ribbon anodes decreases with the distance from the connection points of the supply cable.

Hanif et al.^4^ investigated the external cathodic protection of the above-ground storage tank bottom by using the ribbon mesh grid system. They describe the design process by providing a practical example and correcting NACE's approach for potential shift calculations. In their research, with the help of design parameters, they concluded that the increase in the sand resistivity, anode depth, and anodic current has a direct, inverse, and direct effect on the potential of the tank bottom, respectively. They also provided brief explanations about the effect of oxygen content, leakage monitoring, and the role of the geo membrane layer.

Halafawi et al.^9^ investigated the external (under the tank) and internal cathodic protection of the oil storage tank by using sacrificial zinc and zinc-aluminum anodes, respectively. In this simulation, using AnodeFlex1500 software, they surveyed the effect of anode burial depth under the tank in the form of sensitivity analysis. Researchers found that the deeper the anode burial, the wider the spacing between anodes across the tank bottom area.

A number of factors, including anode burial depth, sand resistivity, and anode spacing in the form of ribbon anodes, have been examined in these studies; however, oxygen-limiting current density and anodic current have not been examined in any detail. Furthermore, a thorough study of wire anodes has been lacking in previous studies. In previous works, we investigated the 3D simulation of anode beds from the perspective of design and optimization under steady-state conditions^10,11^ and considered the transient nature of the corrosion process with deforming anodes for cathodic protection of irregular geometries^12^.

It is worth noting that despite the importance of cathodic protection, the current standard practice in the industry has remained largely unchanged for the past 50 years, relying on classical calculations and large safety factors, and designs are typically based on basic design parameters, such as bare surface area and electrolyte resistivity, and the engineer's experience. As such, there is a need for more innovative and advanced approaches in the field of above-ground storage tank cathodic protection. In this work, we carried out extensive and comprehensive simulations and comparisons to analyze the electrochemical and economical performances of ribbon and wire anodes. Additionally, we provided design recommendations for mesh grids and concentric rings above-ground storage tank cathodic protection systems. Our aim is to compare key parameters such as oxygen-limiting current density and anodic current along with other effective parameters which has not been done in the literature.

This paper is organized as follows: in the first section, a detailed explanation of how to install and drill in the tank bottom is provided. In the second section, based on the theory and the relations that govern the simulation model, calculations are presented for transformer sizing involving the required current, length of the anode, and total resistance for two different configurations with different numbers of conductor bars and power feed cables in ribbon anodes and one configuration with backfilled wire anodes. An analysis of key parameters, such as spacing between anodes, the depth of the anodes, soil resistance, oxygen-limiting current density, and anodic current, is presented in the third section, which examines the cathodic protection of the tank bottom with ribbons and wire anodes. In the fourth section, the economic calculation related to the sacrificial and impressed current anodes has been done by providing a calculation of the required length of each anode.

## Problem statement, excavation, installation, and configurations

### Problem statement

**Figure 1:**
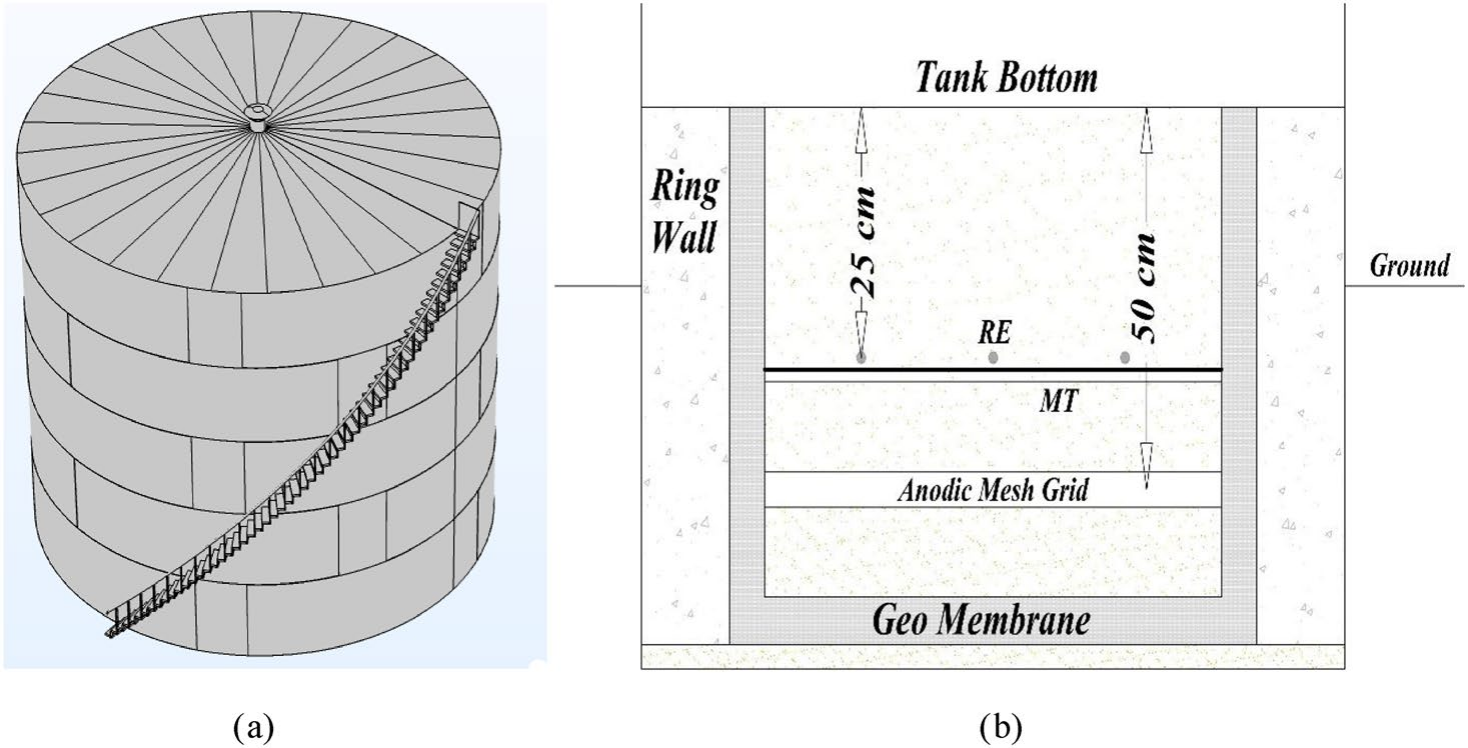
(**a**) Schematic of the storage tank. (**b**) Schematic of installation and drilling along with monitoring tube (MT) and reference electrode (RE).

A cathode protection system design for the outer surface of the above-ground oil storage tank bottom (soil side) is presented with 30 m in diameter and 9 m in height on a foundation of compressed soil with a special electrical resistance of 50 Ω m (Fig. 1a). The bottom surface of the tank is covered with a coal-based epoxy layer before installation and the operating temperature of the tank is 50 °C.

During the welding operation in just the ribbon anode method (at the junction of ribbon anodes with conductor bars), the surrounding area reaches high temperatures and the coating of those areas is somewhat damaged. For this purpose, according to the conditions of using ribbon and wire anodes, a coefficient for coating efficiency has been considered in the calculations. In the simulation of the present study, the welding effect is not presented in order to avoid complexity, but the efficiency factor is considered as an essential recommendation in the design of the tank bottom in the calculations. In the following sections, a brief description of soil contaminants, along with instructions on how to install and operate the protection systems are provided.

### Excavation, and Installation

The soil electrolyte plays a key role in the cathodic protection system and the soil contaminants have a remarkable effect on the rate and type of soil-side corrosion that usually include chlorides, sulfides, and sulfates. Table 1 provides information about the desired properties of sand on the soil-side of the tank bottom according to API 651^2^ and NACE SP0193^13^. These values are recommended for cathodic protection systems.

**Table 1:** Desired properties of sand on the soil-side of tank bottom^14^.

Property	Ac to API 651	Ac to NACE SP0193
Soil resistivity^15^	> 100 $\mathrm{\Omega m}$	> 50 $\mathrm{\Omega m}$
pH^16^	> 8	≥ 5
Chloride levels^17^	< 10 ppm	< 10 ppm
Sulfate levels^18^	< 200 ppm	< 200 ppm
Sulfide levels^19^	< 0.1 ppm	< 0.1 ppm

From installation point of view, see Fig. 1b, after drilling under the tank and sanding on the drilling ground, a concrete ring wall is installed. The ring wall is responsible for keeping the shell because it tolerates the weight of the shell, otherwise the shell wall will fall into the ground and the tank will break down.

In the next step, an insulation layer of 1–6 mm in thickness is created. This insulation layer is commonly made of high-density polyethylene (HDPE). By using the geo membrane layer, the material inside the tank will not enter the ground or the environment if the tank is punctured for any reason (the material inside may be toxic or flammable). Around the tank, there are holes through which the leaking material is drained to the existing pits. To prevent damage to the geo membrane layer, two layers of Geo Textile are used on the top and bottom of the geo membrane with a soft felt-like structure because it is on sand and may be damaged or torn by rocks or unevenness. A layer of sand with water is added uniformly over the sand to ensure that it is well compacted. At a depth of 50 cm to the main bottom, an anode grid is installed. After the anodic grid, sand is poured again.

The reference electrodes are placed at a specific height depending on the dimensions of the tank bottom, the location of anodic mesh grid, the soil resistivity and other environmental factors and have different values. In current case, the reference electrode is placed at a height of about 25 cm to the tank bottom (to find the potential of the bottom of the tank). It is stated in the standard that the number of electrodes reference is tailored to the diameter of the tank^2^ according to Table 2.

**Table 2:** Permanent reference electrodes^2^.

Tank diameter		Number of permanent reference electrodes
m	ft	
6–12	20–40	3
12–18	40–60	4
18–30	60–100	5
30–45	100–150	6
45–76	150–250	7
76–107	250–350	10

**Figure 2:**
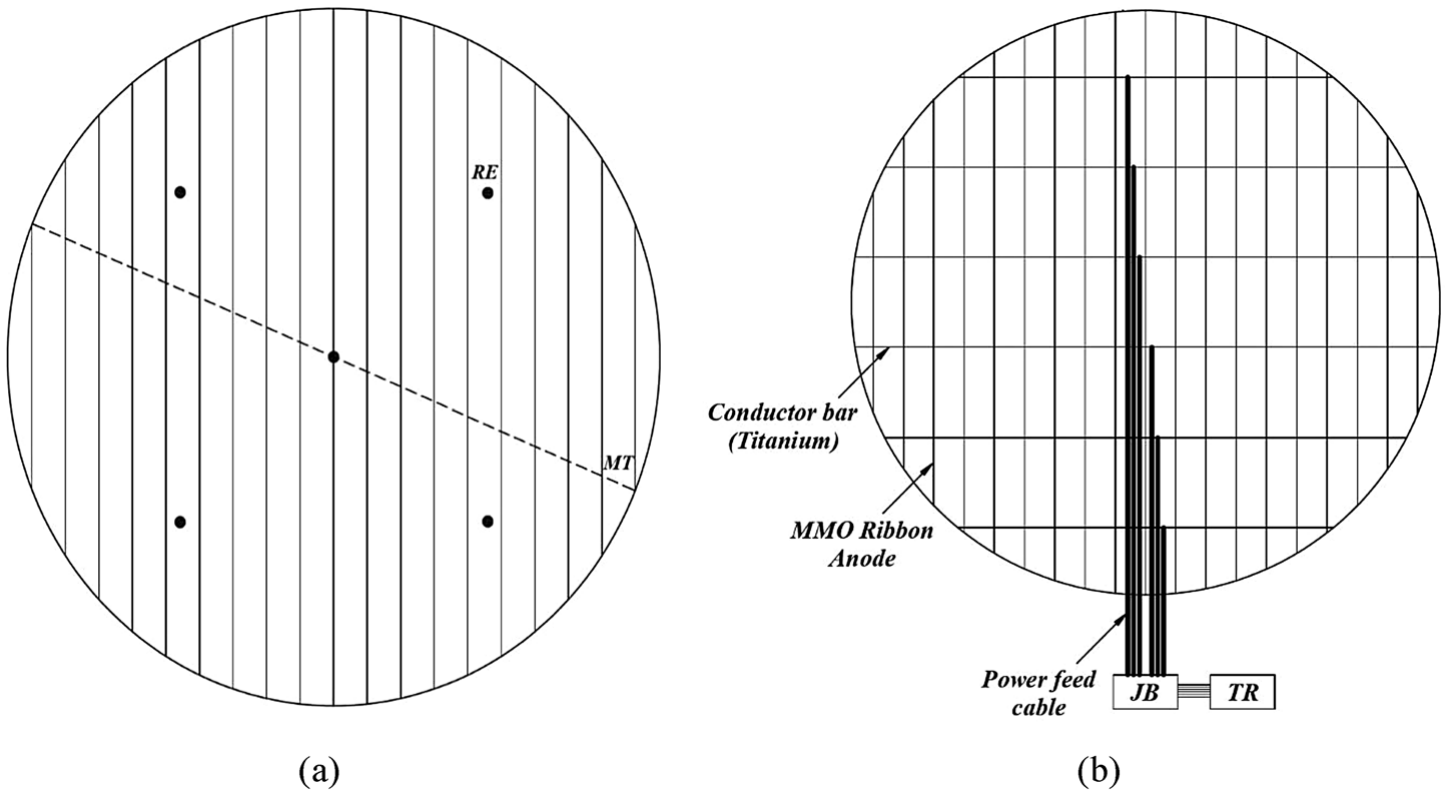
(**a**) Number of reference electrodes to the diameter of the tank and determining the location of the tube monitoring beneath the tank bottom. (**b**) Placing ribbon anodes, titanium bars, and feed cables.

Therefore, according to Table 2, five reference electrodes are required. In Fig. 2a, a reference electrode is placed in the center of the tank, then four reference electrodes with an angle of 90° are placed at two-thirds of the tank radius. The monitoring tube (MT) is also shown in Fig. 2a and junction box (JB) and transformer rectifier (TR) are depicted in Fig. 2b.

The monitoring tube with a 3-inch (7.62 cm) diameter is located by piercing the ring wall at a height of about 25 cm to the bottom of the tank. The reason for using tube monitoring is to send the portable electrode reference under the tank to read the potential of all the points under the tank. The sanding is done again to reach the bottom of the tank.

The bottom of the tank from the outside (soil side) should be painted and this is very critical. When the plates are welded together, part of the paint burns, but a significant portion of the paint remains healthy. This makes the current density required for the entire tank dramatically reduced. Therefore, using a rectifier, can easily transmit the necessary current to places that do not have paint. Companies such as Total, Shell and Aramco, and other well-known companies strongly recommend that the tank bottom be painted^20–23^. It is recommended to use 200 microns of epoxy coal tar or 400 microns bitumen. It is recommended that the distance between the shell and the ring wall be well sealed with substances such as petrolatum. In conditions such as wind, rain, and floods, water does not penetrate the bottom of the tank, as an example, in asphalt bottoms, water is imprisoned on the bottom of the tank (outer floor), resulting in severe damage, as specified by the API standard^2^.

## Configuration of mesh grid and concentric rings systems

Consider a system that has $n$ ribbons, to bring the current to this volume of the ribbon, there are many cables needed, that is, about $n$ cables should be brought under the tank, which is almost not reasonable. To solve this problem, the vertical or conductor bar performs an important task in this anodic grid, so that the conductor bars will reduce the number of cables under the tank. In this case, a certain number of titanium conductor bars perpendicular to MMO ribbons are considered. From an empirical point of view, the interval of the conductor to the conductor is between 2.5 and 5 times (usually 3 times) the ribbon-to-ribbon distance, for example, if the ribbon-to-ribbon distance is 1.5 m, the interval of the conductor to the conductor will be between 3.75 and 7.5 m.

The cables used to connect positively to the anodes and the conductor are made of High Molecular Weight Polyethylene (HMWPE). The cables connected to the conductor bar are called power feed (Fig. 2b). The issue of voltage drop is very common when a power feed cable is used for multiple conductor bars so the number of cables is important.

**Figure 3:**
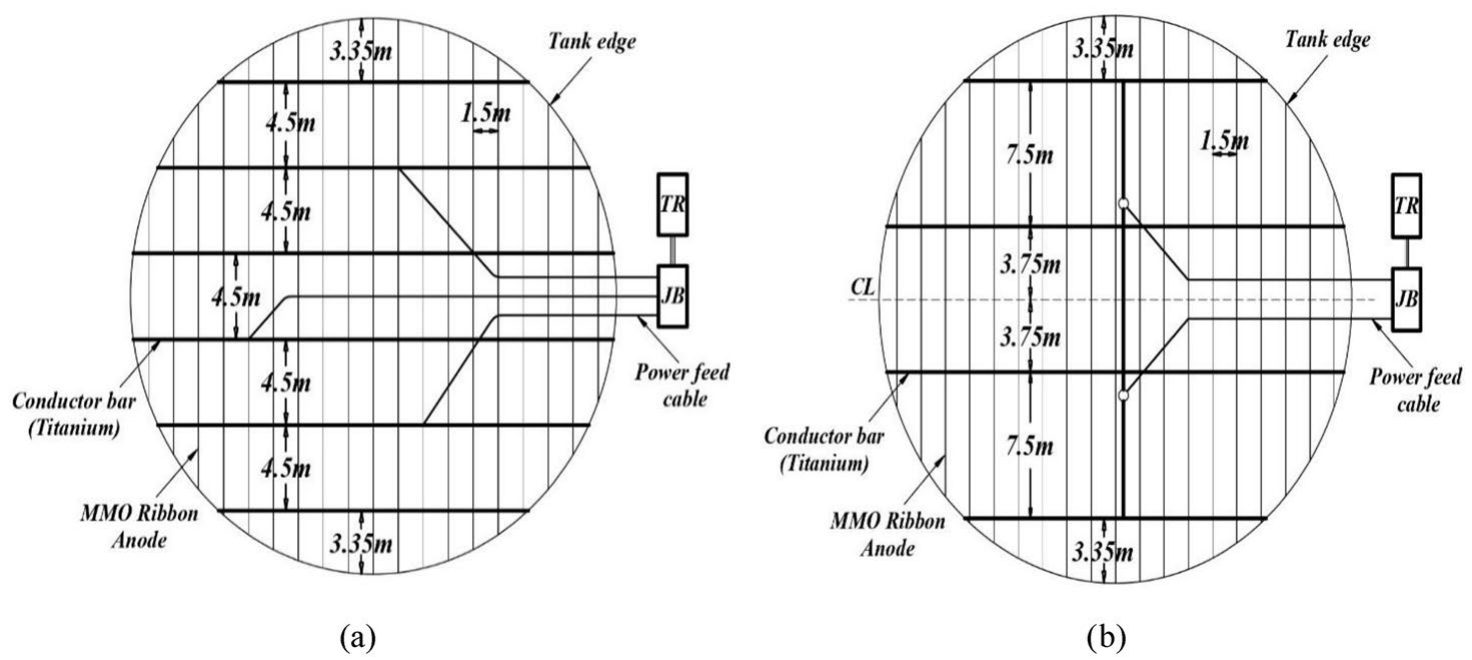
(**a**) Schematic arrangement of 6 conductor bars and 3 power supply cables (first configuration). (**b**) Schematic arrangement of 5 conductor bars and 2 power supply cables (second configuration).

**Figure 4:**
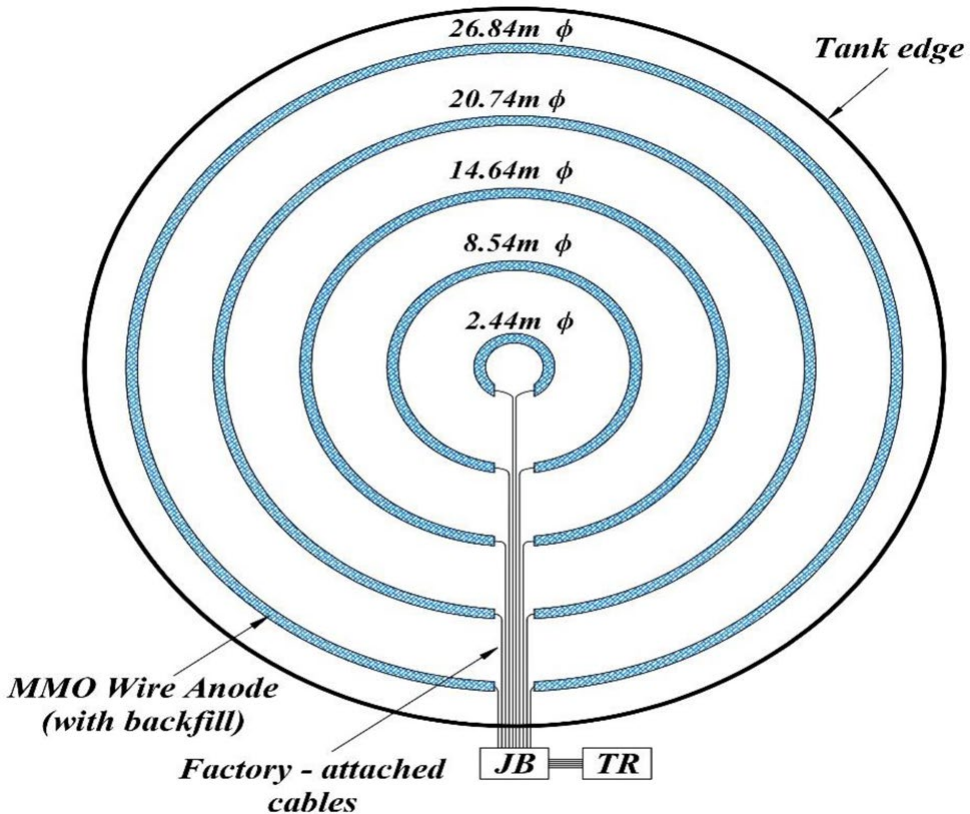
Tank cathodic protection using tank ring anode system for above-ground storage tanks.

For mesh grid system, two configurations are presented, the first configuration includes six conductor bars and three power cables (Fig. 3a), and the second configuration includes five conductor bars and two power cables (Fig. 3b). In the first configuration, the distance between the anodes is 1.5 m and the distance between the conductor bars is 4.5 m, while these values are 1.5 and 7.5 m for the second configuration, respectively. For concentric ring system, a configuration is presented in Fig. 4. Also, explanations about the size of the cables are given and the information related to the resistances in two configurations is shown in Table 6.

These configurations are highly effective in reducing the resistance of the anode grid system, the cost associated with the cables, and better current distribution. According to the calculations, the length of the conductor bars in the first and second configurations is 147 and 117 m, respectively.

## Comparison of mesh grid and concentric rings cathodic protection systems

Despite the long life (over 20 years) of the field-fabricated and field-installed mesh grid anode system, there are a number of deficiencies. With field installation, welding operations, and securing, the ribbon anode and titanium conductor bars have to be field cut to the appropriate lengths. Three spot weld connection of MMO ribbon to titanium conductor bar is applied. The field mesh grid system is subject to weld failures, the spot welding can be damaged easily during subsequent sand installation, and care must be taken to hold the system in place so that it does not short to the tank bottom. All these challenges negatively affect the system performance. In addition, MMO anode (without backfill) in the sand is an oxygen generator when used for cathodic protection. Oxygen is a depolarizer and in some cases, it can lead to problems in maintaining the protection criteria which is proposed in the form of oxygen-limiting current density in the simulation^5^.

In concentric ring systems, anode rings are assembled in the factory and are equipped with the appropriate cable leads while onsite field assembly is not required^5,24,25^. The system requires no splicing, cutting, or welding, and the MMO wire is backfilled within a fabric sleeve with coke breeze^5^. Anode locations are simply marked, each ring is laid out at the proper diameter, and the anode cables are labeled and cabling is extended in the same direction toward the ring wall. In the current system, the presence of coke backfill inhibits the generation of oxygen eliminating the issues with depolarization. Regarding the lifespan, this system is used for more than 30 years, but it can be designed for more than 50 years. It is recommended that for a depth of about one foot (30.5 cm), the spacing between the anodes should be 10 feet (305 cm)^5^. This data has been derived experimentally and validated on numerous actual applications.

## Governing equations

### Electrolytic current

The material balance in the sand soil between anodes and cathode surfaces is expressed as follows:

1 $$(\partial {\mathrm{c}}_{\mathrm{i}}/\partial \mathrm{t})+\nabla \cdot {\mathbf{N}}_{\mathrm{i}}=0$$

where $t$(s) is time, ${c}_{i}$ ($\mathrm{mol}/{\mathrm{m}}^{3}$) is the concentration of $i$ species in the electrolyte and ${{\varvec{N}}}_{i}$ ($\mathrm{mol}/{\mathrm{m}}^{2}\mathrm{s}$) denotes the mass flux vector of $i$ species. By employing the Nernst–Planck relationship and applying logical assumptions, we will arrive at Laplace's equation as follows^26^:

2 $${\nabla }^{2}\mathrm{\varphi }=0$$

where $\varphi (V)$ is the electric potential in the electrolyte. To understand how to arrive at this equation, refer to the Refs.^10,11^. Also, the current density in the electrolyte can be expressed as follows^27^:

3 $$\mathbf{i}=\upsigma \nabla \mathrm{\varphi }$$

where $\mathbf{i}$ ($A/{m}^{2}$) is the current density in the electrolyte and $\sigma \left(S/m\right) is$ the conductivity of the electrolyte.

### Area, current required, the minimum length, and potential shift

The area of protective structure, the tank bottom, and the current required to protect are calculated as follows:

4 $$\mathrm{A}={\mathrm{\pi D}}^{2}/4$$

5 $${\mathrm{I}}_{\mathrm{D}}={i}_{b}{\prime}\times \mathrm{A}\times \left(1-\upxi \right)+{i}_{c}{\prime}\times \mathrm{A}\times\upxi$$

where D ($m$) is diameter of tank, ${i}_{b}{\prime}$($\mathrm{mA}/{\mathrm{m}}^{2}$), $\mathrm{A }({\mathrm{m}}^{2})$, $\upxi$ and ${i}_{c}{\prime} (\mathrm{mA}/{\mathrm{m}}^{2})$ are current density of bare area, surface area, coefficient of coating efficiency and current density of coated area, respectively. Considering that higher currents are needed at high temperatures, for every 10 °C increase in temperature above 30 °C, the current increases by 25% and is expressed as follows^28^:

6 $${\mathrm{I}}_{\mathrm{D},\mathrm{T}}={\mathrm{I}}_{\mathrm{D}}\times {1.25}^{\left(\frac{\mathrm{T}(^\circ \mathrm{C})-30}{10}\right)}$$

where T ($^\circ \mathrm{C}$) is temperature of tank. Even assuming isolation, due to the current leakage, the maximum current requirement is taken into account by $\mathrm{\alpha }$ % of the design margin:

7 $${\mathrm{I}}_{\mathrm{CP}}={\mathrm{I}}_{\mathrm{D},\mathrm{T}}\times (1+\mathrm{\alpha })$$

The area of one meter in the length of the linear anodes with a rectangular (ribbon) and circular (wire) cross-section are calculated from Eqs. (8) and (9), respectively:

8 $${\mathrm{S}}_{\mathrm{An},\mathrm{l},\mathrm{R}}=2\times \left({\mathrm{b}}_{1}+{\mathrm{h}}_{1}\right)$$

9 $${\mathrm{S}}_{\mathrm{An},\mathrm{l},\mathrm{w}}=\uppi \times {\mathrm{d}}_{2}$$

where ${b}_{1}$(mm) and ${h}_{1}$ (mm) are the dimensions of the ribbon anode and ${\mathrm{d}}_{2}$ (cm) is diameter of wire anode. Therefore, the current capacity of each meter of the anode length is counted as follows:

10 $${\mathrm{I}}_{\mathrm{An}}={\mathrm{i}}_{\mathrm{MMO}}\times {\mathrm{S}}_{\mathrm{An},\mathrm{l}}$$

And finally, the minimum value of anode required is calculated by the following relationship:

11 $${\mathrm{L}}_{\mathrm{An},\mathrm{min}}={\mathrm{I}}_{\mathrm{cp}}/{\mathrm{I}}_{\mathrm{An}}$$

**Figure 5:**
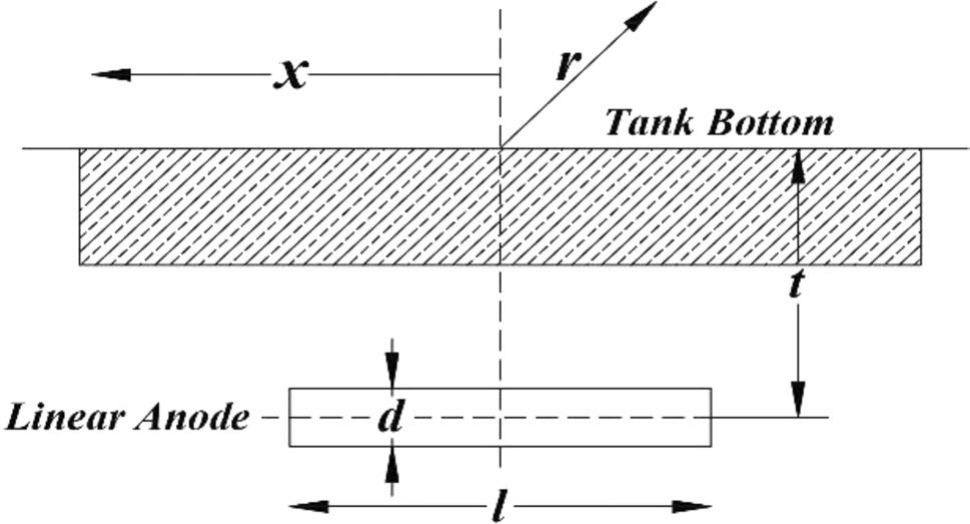
Schematic related to Rodenberg's relationship for near-bed and ribbon anodes in the soil.

In this case, a close bed was created for the tank bottom where the potential shift in adjacent soil with the bottom surface of the tank would be important. Thus, the potential shift in the nearest and farthest point of the tank bottom should be calculated. The farthest point is the distance between ribbon anodes and nearest is above the anode. According to NACE formula with calculating voltage gradient for linear anode of tank bottom (Fig. 5), the potential shift is estimated by the Rodenberg relationship as follow^29^:

12a $${V}_{r, AA}=\frac{\uprho .{\mathrm{I}}_{\mathrm{An}}}{2\times\uppi \times \mathrm{l}}\mathrm{ Ln }\frac{\sqrt{{r}_{min}^{2}+{\mathrm{t}}^{2}+({\mathrm{l}/2)}^{2}}+\mathrm{l}/2}{\sqrt{{r}_{min}^{2}+{\mathrm{t}}^{2}+({\mathrm{l}/2)}^{2}}-\mathrm{l}/2}+{V}_{n}, {r}_{min}=t$$

12b $${V}_{r,MP}=\frac{\uprho .{\mathrm{I}}_{\mathrm{An}}}{2\times\uppi \times \mathrm{l}}\mathrm{ Ln }\frac{\sqrt{{r}_{max}^{2}+{\mathrm{t}}^{2}+({\mathrm{l}/2)}^{2}}+\mathrm{l}/2}{\sqrt{{r}_{max}^{2}+{\mathrm{t}}^{2}+({\mathrm{l}/2)}^{2}}-\mathrm{l}/2}+{V}_{n}, {r}_{max}=\sqrt{{t}^{2}+{\left(Sp/2\right)}^{2}}$$

where $\uprho$ ($\Omega \mathrm{m}$), $\mathrm{l}$ (m), r (m), Sp (m) and t (m) are soil resistivity, length of anode, anode distance to tank bottom, anode spacing and anode depth, respectively. Also ${V}_{n}$, ${V}_{r,AA}$ and ${V}_{r,MP}$ are native potential, potential shift in above the anode and mid-point, respectively. Usually, the lowest potential is recorded at the edges of the tank due to their vulnerability and curvature (especially edges located in the shorter anodes). If the potential at these points is proportional (near) to other parts of the tank, it can be said that the potential of ${V}_{r,AA}$ is the same as the potential of ${V}_{r,Max}$ and potential of ${V}_{r,MP}$ is the same as the potential of ${V}_{r,Min}$.

According to the NACE SP0169^30^, the potential values to confirm the cathodic protection are in the range between − 850 and − 1150 mV with respect to ${\mathrm{Cu}/\mathrm{CuSO}}_{4}$ reference electrode. Also minimum 100 mV of polarization shift as conservative approach is mentioned^4^.

According to NACE, the (13a) equation calculates the maximum spacing between anodes to fulfill the current distribution requirement^29^:

13a $${\mathrm{S}}_{\mathrm{a}}=2 \times \mathrm{t}\times \mathrm{tan }60$$

13b $${\mathrm{S}}_{\mathrm{a}}=2 \times \mathrm{t}\times \mathrm{tan }78.7$$

where $\mathrm{t }(m)$ is anode depth from tank bottom. Of course, Matcor company has used relationship (13b) instead of relation (13a) and stated that the reason for this is to perform many tests on numerous tanks^5^ and in this way, the relation (13b) has been achieved. Assuming the installation of an anodic grid at 50 cm deep (from the tank bottom) with respect to the last layer of soil and 1.5 m distance from each other in ribbon anodes and 3.05 m in wire anodes (a reasonable and conservative choice), the design process continues.

### System resistance

The total resistance of the equivalent circuit is calculated as follows^31^:

14 $${\mathrm{R}}_{\mathrm{eq}}={\mathrm{R}}_{\mathrm{An},\mathrm{in}}+{\mathrm{R}}_{\mathrm{An},\mathrm{F}}+{\mathrm{R}}_{{\mathrm{C}}^{+}}+{\mathrm{R}}_{{\mathrm{C}}^{-}}+{\mathrm{R}}_{\mathrm{st},\mathrm{e}}+{\mathrm{R}}_{\mathrm{st},\mathrm{in}}+{\mathrm{R}}_{\mathrm{An},\mathrm{e}}$$

where ${\mathrm{R}}_{\mathrm{An},\mathrm{in}}$ is resistance of mesh grid anode or concentric ring anode, ${\mathrm{R}}_{\mathrm{An},\mathrm{F}}$ is resistance of power feed cable, ${\mathrm{R}}_{{\mathrm{C}}^{+}}$ and ${\mathrm{R}}_{{\mathrm{C}}^{-}}$ are positive and negative cable resistance, ${\mathrm{R}}_{\mathrm{st},\mathrm{e}}$, ${\mathrm{R}}_{\mathrm{st}}$ and ${\mathrm{R}}_{\mathrm{An},\mathrm{e}}$ are structural resistance to the electrolyte, internal resistance of the tank bottom and anode resistance to the electrolyte. In the following, the resistance of each part is explained.

#### Anode resistance to electrolyte (${R}_{An,e}$)

##### Ribbon

The presence of the geo membrane layer, parallel configuration of the ribbon anodes and the proximity of the mesh grid system to the tank bottom complicate the evaluation.

The main purpose of this section is to calculate the resistance of the anode to the soil electrolyte, in which an approximate method called Dwight's modified method is used, which is somewhat reliable. Assuming the average length of ribbon anode and using Dwight's modified formula^31,32^, the average ribbon anode resistance is obtained as follows and Table 3 defines corresponding parameters:

**Table 3:** Definition of parameters for calculating resistance of average ribbon anode.

$R=\left({R}_{1}+{R}_{2}\right)/2 (\Omega )$	$\rho$($\mathrm{\Omega m}$)	L (m)	${d}_{eq}$(m)	$\theta$	S or t (m)
Resistance of average ribbon	Soil resistivity	Average length of ribbon	Eq diameter of ribbon	Angle bt bottom and ribbon	depth of ribbon
–	50	$446/19=$ 23.48	0.0045	38.7°	0.5

15 $${\mathrm{R}}_{1}=\frac{\uprho }{2\mathrm{\pi L}} \left[\mathrm{ln}\left(\frac{4\mathrm{L}}{{d}_{eq}}\right)+\mathrm{ln}\left(\frac{\mathrm{L}}{\mathrm{S}}\right)-2+\left(\frac{2\mathrm{S}}{\mathrm{L}}\right)\right]$$

**Figure 6:**
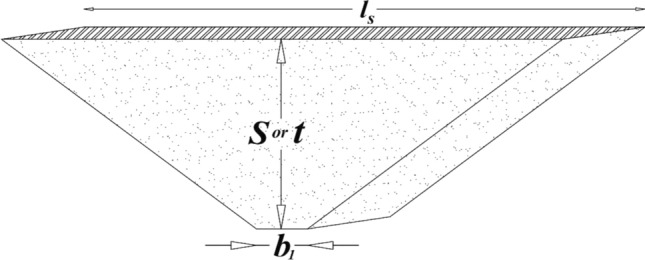
Schematic of the tank bottom and ribbon anode to calculate the resistance ${R}_{2}$.

According to Fig. 6 and by developing an equation for a single anode strip and assuming ${b}_{1}\ll S$ , resistance of anode with average length can be evaluated as follows^31^:

16 $${R}_{2}=\frac{ \rho }{2L tan\theta }\mathit{ln}\left(\frac{2S tan\theta }{{b}_{1}}\right)$$

To determine the equivalent radius ($${r}_{eq})$$ if the cross-section of the anode is not circular, the following equation is used^33,34^:

17 $${r}_{eq}=C/2\pi$$

where C is considered as the perimeter of the cross-section, which is calculated as follows in the rectangular cross-section:

18 $${r}_{eq}=2({b}_{1}+{h}_{1})/2\pi{\qquad or\qquad } {d}_{eq}=2({b}_{1}+{h}_{1})/\pi$$

To calculate the equivalent length, dividing the length of the required MMO ribbon anode by the number of ribbon anodes (i.e. 19) is used.

To calculate the resistance value more rationally, the average of two values ${R}_{1}$ and ${R}_{2}$ is used^31^. In this study, the value of anode grid resistance (R) is calculated as 0.28 $\Omega$.

##### Wire

The calculations related to the resistance of the wire anode to the soil can be calculated from the modified Dwight relation and for the circular section.

Each anode ring is connected parallel to one another through the anode junction box to the rectifier. The resistance of each anode ring is calculated using Dwight’s equation for a ring of wire^5,32^ and Table 4 defines corresponding parameters:

**Table 4:** Definition of parameters for calculating resistance of each ring.

R ($\Omega$)	$\uprho$ ($\mathrm{\Omega m}$)	${\mathrm{D}}^{\mathrm{^{\prime}}}$ (m)	${\mathrm{d}}_{2}$ (cm)	${\mathrm{s}}^{\mathrm{^{\prime}}}$ (cm)
Resistance of each ring	Soil resistivity	Diameter of ring	Diameter of anode	Twice the depth of anode
–	50	2.44, 8.54, 14.64, 20.74, 26.84	1.27	100

19 $$\mathrm{R}=\frac{\uprho }{2{\uppi }^{2}{\mathrm{D}}^{\mathrm{^{\prime}}}} \left({\mathrm{log}}_{\mathrm{n}}8{\mathrm{D}}^{\mathrm{^{\prime}}}/{\mathrm{d}}_{2}+{\mathrm{log}}_{\mathrm{n}}4{\mathrm{D}}^{\mathrm{^{\prime}}}/{\mathrm{s}}^{\mathrm{^{\prime}}}\right)$$

Each ring’s resistance is calculated and then the total resistance is calculated as^21,31^

20 $$\frac{1}{{\mathrm{R}}_{\mathrm{T}}}=\frac{1}{{\mathrm{R}}_{1}}+\frac{1}{{\mathrm{R}}_{2}}+\frac{1}{{\mathrm{R}}_{3}}+\dots +\frac{1}{{\mathrm{R}}_{\mathrm{n}}}$$

It is important to note that the resistivity directly impacts the resistance calculations and often the actual sand resistivity can vary from the design basis affecting the resistance calculations and rectifier sizing.

#### Anode internal resistance (${R}_{An,in}$)

When the area is very small compared to the length of the anode, the value of the internal resistance of the anode becomes important and noticeable. In this part, due to the small dimensions of linear anodes, their internal resistance has been calculated.

##### Ribbon

In use of ribbon anodes, there is a problem with current or potential attenuation. It is noteworthy that the internal resistance of the volume anodes (e.g. cylindrical) can be avoided, while for longitudinal or linear anodes such as ribbon or wire anodes, internal resistance is high and must be reduced in some way. In the case of the anodic grid system, this problem is fixed by titanium conductor bars that are perpendicular to the ribbon anodes. These titanium bars reduce the longitudinal resistance of the MMO ribbon mesh to distribute the current uniformly and are connected to all parts of the surface of the tank bottom using point welding.

Ribbon anodes have different dimensions, but usually, some dimensions are more commercially produced. In this section, one of the conventional dimensions is used. These anodes are with ${\mathrm{b}}_{1}$ width and ${\mathrm{h}}_{1}$ in thick and titanium conductor bars are made of titanium with ${\mathrm{b}}_{1}^{\mathrm{^{\prime}}}$ width and ${\mathrm{h}}_{1}^{\mathrm{^{\prime}}}$ in thick. These values are given in Table 8.

The resistance of each MMO anode cell and titanium bar cell is simply calculated. It is noteworthy that both the MMO anode and the titanium bars have titanium structure, so the same resistivity^35^ can be 0.42 $(\mathrm{\mu \Omega m})$ and the calculations are as follows:

21 $${\mathrm{R}}_{\mathrm{An},\mathrm{Cell}}={\uprho }_{\mathrm{Ti}} \frac{{\mathrm{l}}_{\mathrm{An},\mathrm{Cell}}}{{\mathrm{A}}_{\mathrm{An}}}={\uprho }_{\mathrm{Ti}} \times \frac{{\mathrm{S}}_{1}^{\mathrm{^{\prime}}}}{{\mathrm{b}}_{1}\times {\mathrm{h}}_{1}}$$

22 $${\mathrm{R}}_{\mathrm{Ti},\mathrm{Cell}}={\uprho }_{\mathrm{Ti}} \frac{{\mathrm{l}}_{\mathrm{Ti},\mathrm{Cell}}}{{\mathrm{A}}_{\mathrm{Ti}}}={\uprho }_{\mathrm{Ti}} \times \frac{{\mathrm{S}}_{1}}{{{\mathrm{b}}_{1}}^{\mathrm{^{\prime}}}\times {{\mathrm{h}}_{1}}^{\mathrm{^{\prime}}}}$$

where ${\uprho }_{\mathrm{Ti}}$, ${\mathrm{l}}_{\mathrm{An},\mathrm{Cell}}$, ${\mathrm{S}}_{1}^{\mathrm{^{\prime}}}$, ${\mathrm{S}}_{1}$, ${{\mathrm{b}}_{1}}^{\mathrm{^{\prime}}}$ and ${{\mathrm{h}}_{1}}^{\mathrm{^{\prime}}}$ are resistivity of titanium, anode cell length, distance of conductor to conductor, distance of anode to anode and dimension of conductor bars, respectively. Calculations of the anode internal resistance or ribbon grid are complex. With the development of the anode grid and the increase of these cells, the overall resistance of the grid decreases. By carrying out industrial projects and the experience gained from them, in order to calculate the internal resistance of the anode grid, relation (23) has been proposed. In fact, as this grid grows larger and larger, the coefficient number tends to $\sqrt{3}$. In this case, the max grid resistance can be calculated as follows, so that the value mentioned must be multiplied by the average of two cells:

23 $${\mathrm{R}}_{\mathrm{Grid},\mathrm{ max}}= \sqrt{3}\times \left({\mathrm{R}}_{\mathrm{An},\mathrm{Cell}}+{\mathrm{R}}_{\mathrm{Ti},\mathrm{Cell}}\right)/2$$

The number of conductor bars and power feed cables will play an effective role in calculating the internal resistance of ribbon anodes which is presented in Fig. 3.

##### Wire

Similar to Eq. (21), the internal resistance of each of the wire anodes is calculated as follows:

24 $${\mathrm{R}}_{\mathrm{An},\mathrm{Ring}}={\uprho }_{\mathrm{Ti}} \frac{{\mathrm{l}}_{\mathrm{An},\mathrm{Ring}}}{{\mathrm{A}}_{\mathrm{An}}}={\uprho }_{\mathrm{Ti}} \frac{\uppi \times {\mathrm{D}}^{\mathrm{^{\prime}}} }{\left(\uppi /4\right){\mathrm{d}}_{2}^{2}}={\uprho }_{\mathrm{Ti}} \frac{4{\mathrm{D}}^{\mathrm{^{\prime}}} }{{\mathrm{d}}_{2}^{2}}$$

Equation (20) is used to calculate the overall internal resistance of wire anodes. The results related to the internal resistance of the system, anode resistance to electrolyte and calculations associated with lengths are presented in Table 5.

**Table 5:** Detailed data about dimensional parameters and resistance of each ring.

Ring	Diameter (m)	Length wire anode (m)	Tail cable length (m)	${\mathrm{R}}_{\mathrm{An},\mathrm{e}}$ ($\Omega$)	${\mathrm{R}}_{\mathrm{An},\mathrm{in}}$ ($\Omega$)
1	2.44	2.44 $\uppi$	39.04	9.97	0.026
2	8.54	8.54 $\uppi$	32.94	3.59	0.089
3	14.64	14.64 $\uppi$	26.84	2.28	0.153
4	20.74	20.74 $\uppi$	20.74	1.69	0.216
5	26.84	26.84 $\uppi$	14.64	1.36	0.28
Total		230	134	0.467	0.016

By looking at Table 5, the values of the length of the wire anode, the length of the tail cable, the resistance of the anode to the electrolyte, and the internal resistance of the anode have been calculated at 230 m, 134 m, 0.467 $\Omega$, and 0.016 $\Omega$, respectively. As expected, the effect of internal resistance is negligible in ring-backfilled wire anodes, while this value is significant in grid ribbon anodes. The use of a coating containing backfill (compacted coke) around the wire anode, in addition to reducing the amount of oxygen, has increased the diameter of the anode. The increase in diameter, in addition to more current, reduces the internal resistance of the anode and reduces the required voltage or the sizing of the transformer rectifier.

#### Other resistances and equivalent resistance

If a large amount of cables is needed, the resistance of the cables becomes important. The specifications of the cables in different configurations are as follows:

In the first configuration, three feed cables of 1 × 10 ${\mathrm{mm}}^{2}$ to uniform current distribution and circuit resistance reduction and each to 50 m length are provided. One main positive cable with 1 × 35 ${\mathrm{mm}}^{2}$ size and 100 m length and 1 main negative cable (connecting cable to the body of the tank) with a size of 1 × 35 ${\mathrm{mm}}^{2}$ and a length of 120 m are utilized. In the second configuration, two feed cables of 1 × 10 ${\mathrm{mm}}^{2}$ with an average length of 50 m, main positive and negative cables with 1 × 35 ${\mathrm{mm}}^{2}$ size with length of 80 and 120 m are used.

In wire configuration and according to Fig. 4, five tail cables of 1 × 10 ${\mathrm{mm}}^{2}$ and each to 27 m average length are provided. Also, one main positive cable with 1 × 35 ${\mathrm{mm}}^{2}$ size and 80 m length and one main negative cable with a size of 1 × 35 ${\mathrm{mm}}^{2}$ and a length of 120 m are utilized.

In the above-ground tank anodic grid system and concentric rings, the main resistances are cable resistance, internal resistance (grid), and anode resistance to the electrolyte while the structural resistance to the electrolyte can be ignored due to the larger surface of the structure, as well as the internal resistance of the structure, namely the internal resistance of the tank bottom, due to the very large surface and very small length (thickness of the tank bottom), can be neglected. In this article, the sum of the resistance ${\mathrm{R}}_{\mathrm{st},\mathrm{e}}$ and ${\mathrm{R}}_{\mathrm{st}}$ is considered equal to 0.05 $\Omega$. Information related to the values of resistances in different sections is presented in Table 6.

**Table 6:** Calculation of resistance of different components in three investigated configurations.

Type/unit ($\Omega$)	${\mathrm{R}}_{\mathrm{An},\mathrm{in}}$	${\mathrm{R}}_{\mathrm{An},\mathrm{F}}$	${\mathrm{R}}_{{\mathrm{C}}^{+}}$	${\mathrm{R}}_{{\mathrm{C}}^{-}}$	${\mathrm{R}}_{\mathrm{An},\mathrm{e}}$	${\mathrm{R}}_{\mathrm{st},\mathrm{e}}+{\mathrm{R}}_{\mathrm{st}}$	${\mathrm{R}}_{\mathrm{eq}}$
${\mathrm{Ribbon}}_{\mathrm{C}1}$	0.152	0.031	0.052	0.063	0.280	0.05	0.628
${\mathrm{Ribbon}}_{\mathrm{C}2}$	0.362	0.046	0.035	0.063	0.280	0.05	0.836
Wire	0.016	0.010	0.042	0.063	0.467	0.05	0.648

In the anode network system, the values of the anode internal resistance and the resistance of the anode to the electrolyte are the first priority and the resistance of the cables is the second priority. While in the concentric ring model, only the resistance of the anode to the electrolyte has been calculated as the dominant resistance, and other resistances are the next priority.

## Selection of appropriate transformer rectifier

It is very important to choose the right transformer rectifier along with the related sizing, the desired voltage, and current and it must be chosen according to the climate. With a simple calculation, we can say that with having the total resistance (according to Table 6) and current needed for protection, the output voltage required for the cathode protection considering back voltage potential by 2V is calculated as follows:

25a $${E}_{cp,R,{C}_{1}}={I}_{cp}\times {R}_{eq}+{E}_{b}=17.6\times 0.628+2=13.05\mathrm{ V}$$

25b $${E}_{cp,R,{C}_{2}}={I}_{cp}\times {R}_{eq}+{E}_{b}=17.6\times 0.836+2=16.71\mathrm{ V}$$

25c $${E}_{cp,R}={I}_{cp}\times {R}_{eq}+{E}_{b}=12.3\times 0.648+2=10.0\mathrm{ V}$$

Therefore, for a cathodic protection system with an impressed current method, an anodic grid can be used by MMO ribbon anodes at 1.5 m spacing and a rectifier with a minimum current of 17.6 A and a voltage of 13 V in the first configuration and 16.7 in the second configuration must be utilized. Also, a concentric ring can be utilized by MMO wire anodes at 3.05 m intervals and a rectifier with a minimum current of 12.3A and a voltage of 10.0 V must be used.

As the calculations show, choosing a concentric ring system with lower voltage and current will put less pressure on the transformer and smaller sizing can be used as well. In all three configurations, one of the choices is the use of a rectifier with a voltage output capacity of 20 A and 20 V.

## Conditions and parameters

### Boundary conditions

In order to solve Eqs. (2) and (3) in the computing domain, we need to use related boundary conditions in the form of Tafel relations. Tafel's relations, which relate electrochemical parameters, are used as boundary conditions for anode and cathode as follows^27^:

26 $${\mathrm{i}}_{\mathrm{a}}\left(\mathrm{\varphi }\right)={\mathrm{i}}_{0,\mathrm{a}}\times {10}^{\frac{{\upeta }_{\mathrm{a}}\left(\mathrm{\varphi }\right)}{{\upbeta }_{\mathrm{a}}}}\mathrm{\qquad and\qquad }{\mathrm{i}}_{\mathrm{c}}\left(\mathrm{\varphi }\right)={\mathrm{i}}_{0,\mathrm{c}}\times {10}^{\frac{{\upeta }_{\mathrm{c}}\left(\mathrm{\varphi }\right)}{{\upbeta }_{\mathrm{c}}}}$$

where i ($\mathrm{mA}/{\mathrm{m}}^{2}$), ${\mathrm{i}}_{0}$ ($\mathrm{mA}/{\mathrm{m}}^{2}$), and $\upbeta$ (mV) are the current density, exchange current density, and Tafel slope, respectively. $\upeta \left(\mathrm{mV}\right)$ is the over-potential and it is defined as follows^27^:

27 $${\upeta }_{\mathrm{a}}={\mathrm{E}}_{\mathrm{a}}-{\mathrm{E}}_{\mathrm{eq},\mathrm{a}}\mathrm{\qquad and \qquad }{\upeta }_{\mathrm{c}}={\mathrm{E}}_{\mathrm{c}}-{\mathrm{E}}_{\mathrm{eq},\mathrm{c}}\mathrm{\qquad and \qquad E}=\mathrm{ \varphi }-{\mathrm{\varphi }}_{\mathrm{e}}$$

where $\mathrm{\varphi }$ and ${\mathrm{E}}_{\mathrm{eq}}$ are potential and equilibrium potential of electrodes, respectively and the subscripts *a*, *c*, and *e* correspond to the anode, cathode, and electrolyte. The changes of Tafel parameters depend on the electrode materials and electrolyte properties. By integrating the above equations, the general form of Butler–Volmer equations for the tank bottom is obtained as follows^36–38^:

28 $$\mathrm{i}={\mathrm{i}}_{\mathrm{a}}-{\mathrm{i}}_{\mathrm{c}1}-{\mathrm{i}}_{\mathrm{c}2}={\mathrm{i}}_{0,\mathrm{Fe}}{\times 10}^{\left(\frac{{\mathrm{E}}_{\mathrm{Fe}}-{\mathrm{E}}_{\mathrm{eq},\mathrm{Fe}}}{{\upbeta }_{\mathrm{Fe}}}\right)}-{\mathrm{i}}_{\mathrm{lim},{\mathrm{O}}_{2}}-{\mathrm{i}}_{0,{\mathrm{H}}_{2}}{\times 10}^{\left(\frac{{\mathrm{E}}_{{\mathrm{H}}_{2}}-{\mathrm{E}}_{\mathrm{eq},{\mathrm{H}}_{2}}}{{\upbeta }_{{\mathrm{H}}_{2}}}\right)}$$

where ${\mathrm{i}}_{\mathrm{a}}$, ${\mathrm{i}}_{\mathrm{c}1}$ and ${\mathrm{i}}_{\mathrm{c}2}$ represent the current density generated by iron oxidation, oxygen reduction and hydrogen evolution, respectively. In this regard, the values related to Tafel parameters for MMO ribbon grid system and wire concentric rings arrangements in the tank bottom are presented in Table 7.

**Table 7:** Tafel parameter values for tank bottom and MMO ribbon and wire anode in contact with the sand soil.

Type	${\mathrm{i}}_{0,\mathrm{Fe}}$ ($\mathrm{mA}/{\mathrm{m}}^{2})$	${\mathrm{i}}_{0,{\mathrm{H}}_{2}}$ ($\mathrm{mA}/{\mathrm{m}}^{2})$	${\mathrm{i}}_{\mathrm{lim}, {\mathrm{O}}_{2}}$ ($\mathrm{mA}/{\mathrm{m}}^{2})$	${\mathrm{i}}_{\mathrm{MMO}}$ ($\mathrm{A}/{\mathrm{m}}^{2})$	${\upbeta }_{\mathrm{Fe}}$ (mV)	${\upbeta }_{{\mathrm{H}}_{2}}$ (mV)	${\mathrm{E}}_{\mathrm{eq},\mathrm{Fe}}$ (V)	${\mathrm{E}}_{\mathrm{eq},{\mathrm{H}}_{2}}$ (V)
Ribbon	10	0.2	10	3	60	− 120	− 0.55	− 0.73
Wire	5	0.1	5	1.5	60	− 120	− 0.55	− 0.73

In the presented simulation, the effect of oxygen limiting current density (${\mathrm{i}}_{{\mathrm{lim},\mathrm{o}}_{2}}$) along with hydrogen current density (${\mathrm{i}}_{{0,\mathrm{H}}_{2}}$) and MMO anode (${\mathrm{i}}_{\mathrm{MMO}}$) is more noticeable and has been given more attention. In this way, by numerically solving the computational grid and using Tafel boundary conditions, the distribution of current density and potential in the computational domain is obtained. In the case of ${\mathrm{i}}_{\mathrm{MMO}}$, the values considered are different depending on the conditions and are selected from the catalog of the companies.

### Input and output parameters

Table 8 presents the input and output parameters related to the governing equations along with number of equation (N.E). In the present model, index 1 or R is used for ribbon anodes and index 2 or W is used for wire anodes.

**Table 8:** Information about parameters related to dimension of bottom of tank and anodes, condition of coating, anodes environment and result from equations.

Input	Value (unit)	N.E	Input	Value (unit)	N.E	Output	Value (unit)	N.E	Output	Value (unit)	N.E
$\mathrm{D}$	30 (m)	**4**	${\mathrm{i}}_{\mathrm{MMO},1}$	3 ($\mathrm{A}/{\mathrm{m}}^{2}$)	**10**	$\mathrm{A}$	707 (${\mathrm{m}}^{2}$)	**4**	${\mathrm{I}}_{\mathrm{An},1}$	0.042 (A)	**10**
${\upxi }_{1}$	55 ($\mathrm{\%}$)	**5**	${\mathrm{i}}_{\mathrm{MMO},2}$	1.5 ($\mathrm{A}/{\mathrm{m}}^{2}$)	**10**	${\mathrm{I}}_{\mathrm{D},1}$	9.39 (A)	**5**	${\mathrm{I}}_{\mathrm{An},2}$	0.060 (A)	**10**
${\upxi }_{2}$	70 ($\mathrm{\%}$)	**5**	${\mathrm{b}}_{1}$	6.35 (mm)	**8,21**	${\mathrm{I}}_{\mathrm{D},2}$	6.56 (A)	**5**	${\mathrm{L}}_{\mathrm{An},\mathrm{min},1}$	419 (m)	**11**
${i}_{b}{\prime}$	28 ($\mathrm{mA}/{\mathrm{m}}^{2}$)	**5**	${\mathrm{h}}_{1}$	0.635 (mm)	**8,21**	${\mathrm{I}}_{\mathrm{D1,50}}$	14.67 (A)	**6**	${\mathrm{L}}_{\mathrm{An},\mathrm{min},2}$	205 (m)	**11**
${i}_{c}{\prime}$	1.25 ($\mathrm{mA}/{\mathrm{m}}^{2}$)	**5**	${\mathrm{d}}_{2}$	1.27 (cm)	**9**	${\mathrm{I}}_{\mathrm{D2,50}}$	10.25 (A)	**6**			
T	50 ($\mathrm{C}$)	**6**	${\mathrm{b}}_{1}^{\mathrm{^{\prime}}}$	12.7 (mm)	**22**	${\mathrm{I}}_{\mathrm{CP},1}$	17.6 (A)	**7**			
$\mathrm{\alpha }$	20 (%)	**7**	${\mathrm{h}}_{1}^{\mathrm{^{\prime}}}$	0.9 (mm)	**22**	${\mathrm{I}}_{\mathrm{CP},2}$	12.3 (A)	**7**			
$\uprho$	50 ($\mathrm{\Omega m})$	**12**	${\mathrm{S}}_{1}$	1.5 (m)	**22**	${\mathrm{S}}_{\mathrm{An},\mathrm{l},\mathrm{R}}$	0.014 ${(\mathrm{m}}^{2})$	**8**			
t	0.5 (m)	**12**	${\mathrm{S}}_{1}^{\mathrm{^{\prime}}}$	4.5 (m)	**21**	${\mathrm{S}}_{\mathrm{An},\mathrm{l},\mathrm{w}}$	0.040 (${\mathrm{m}}^{2})$	**9**			

The information presented in Table 8 is provided by the results of various projects and some reliable sources^4,5,31,39^. The minimum length of linear anode required is 419 m and 205 m; therefore 446 m and 230 m is acceptable for ribbon and wire, respectively. The actual length of the whole MMO ribbon anode is considered to be about 446 m according to the calculations in supplementary material.

## Model validation

**Figure 7:**
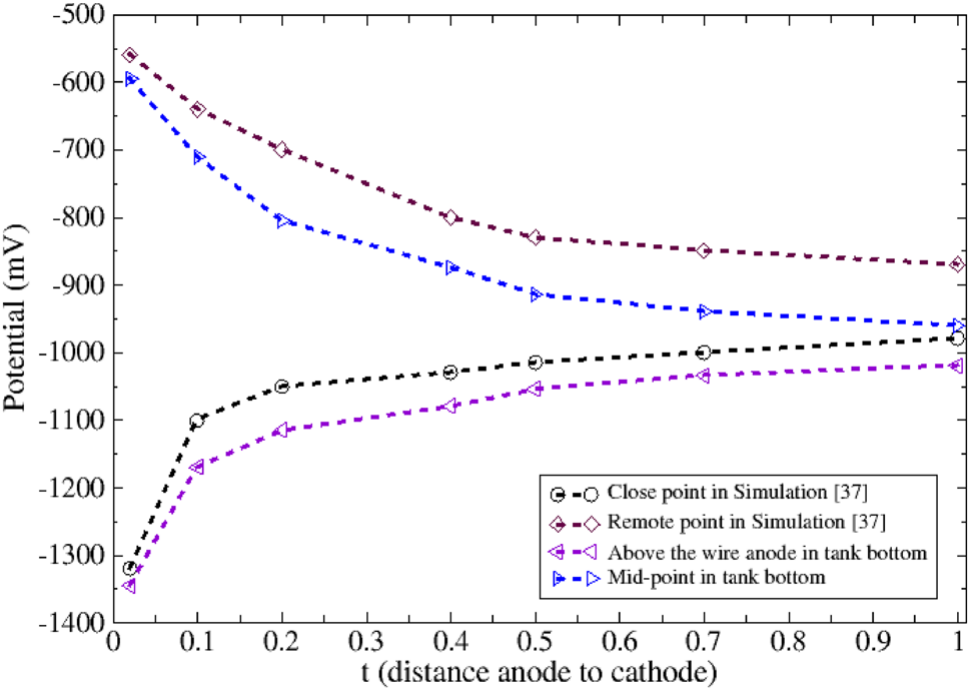
Comparison between the minimum and maximum potential profile of close and remote points of pipe in^37^ and mid-point (min potential) and above the wire anode (max potential) in the current study.

To validate the accuracy of the simulation model, the results from the present study have been compared against those from a similar previous simulation^37^. Both simulations modeled an MMO wire anode with a 3 mm circular cross-section and current density of 3 A/m^2^ (28 mA/m). The difference was in the protected structure—a steel pipe with 0.6 m diameter in^37^ versus a tank bottom in the present study, with an anode spacing of 1.2 m. Other parameters like soil resistivity (20 Ω m) and oxygen limiting current density (20 mA/m^2^) were identical. As shown in Fig. 7, the minimum and maximum potential values at two measurement points relative to cathode distance match closely between the simulations. The minimum potential matched within 9.5% error, while the maximum potential was within 5.5% error. This close agreement is expected given the simulations' similar configuration, with the primary variance being the protected structure. The results validate the model's accuracy in predicting corrosion protection performance across different applications while maintaining comparable soil conditions and protection criteria. Overall, consistency between the findings lends confidence to the model's ability to reasonably simulate cathodic protection system behavior.

## Results and discussions

To accurately simulate the electrochemical process of cathodic protection for tank bottoms, a nonlinear system of governing equations needs to be solved numerically. In this study, we employed COMSOL MULTIPHYSICS (V 5.2), a finite element PDE solver, to effectively solve these equations and derive the distributions of potential and current density within the computational domain. The main solver is PARDISO and non-linear method is selected newton. PARDISO is the acronym of parallel sparse direct solver. Indeed, PARDISO is a thread-safe, high performance, robust, memory efficient and easy to use software for solving large sparse symmetric and asymmetric linear systems of equations on shared memory multi-processors with distributed memory. The linear system solver works on general sparse linear systems of the form $\mathrm{Ax}=\mathrm{b}$ and use LU factorization on the matrix A to compute the solution x. The automatic detection works through analysis of the variables contributing to the residual Jacobian matrix and the constraint Jacobian matrix.

In this section, we begin by providing a concise overview of the mesh size utilized. Subsequently, we delve into a comprehensive discussion of the simulation process for tank bottom protection, focusing on two distinct methods: the mesh grid system and the concentric ring system. Throughout this analysis, we extensively explore key parameters, including sand resistivity, oxygen-limiting current density, anode depth, and anodic current. By thoroughly investigating these factors, we aim to gain valuable insights into the effectiveness of both protection methods.

### Mesh grid system

Given the small dimensions of the ribbon anodes, we have employed an extra fine element size in the meshing process. The resulting meshes comprise 491,785 mesh vertices, 2,772,513 tetrahedron elements, 148,319 triangle elements, 36,570 edge elements, and 255 vertex elements, with an average element quality of 0.8205. With the completion of this meshing process, we will now proceed with discussing the continuation of the simulation process. The potential and current density distribution depend on the distance between the anodes, the soil resistance, the depth of burial anodes from the tank bottom, the length of the anode required, and the oxygen-limiting current density etc. In the following sections, the key parameters are examined.

#### Spacing between the anodes

**Figure 8:**
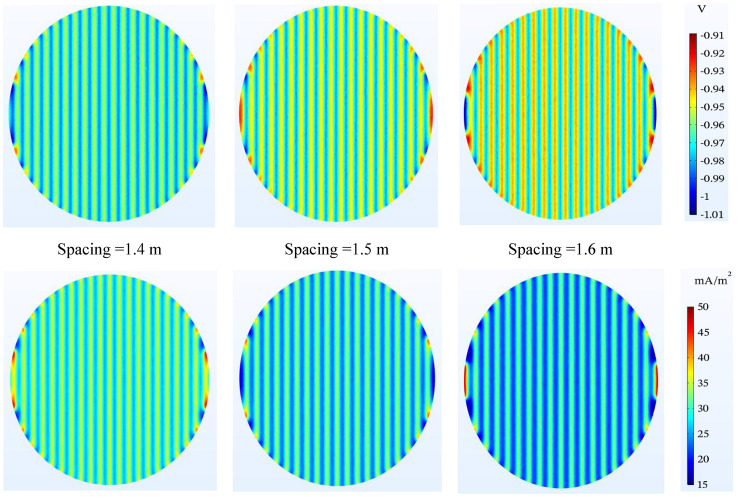
Potential ($\mathrm{V}-\mathrm{CSE}$) and current density ($\mathrm{mA}/{\mathrm{m}}^{2}$) distribution on the tank bottom at 0.5 m to bottom plate and different spacing between ribbons.

According to the simulation results, the closer the anodes are to each other, the better the tank bottom will be protected. It is worth noting that in all three cases, the tank bottom potential confirms the protection criterion. A smaller distance between the anodes causes more ribbons to be used, which increases the cost. In this study, a distance of 1.5 m is considered as a good criterion. Of course, for a distance of 1.4 m, the bottom of the tank receives more current density which can be clearly seen in Fig. 8.

**Figure 9:**
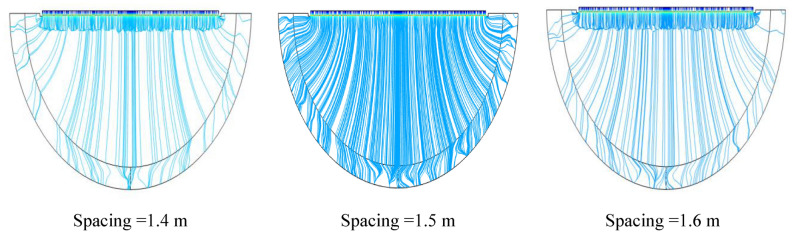
Streamlines of cathodic protection current at 0.5 m to tank bottom and different spacing between ribbons.

As can be seen in Fig. 9, for a spacing of 1.5 m, the streamlined distribution is displayed proportionally and more uniformly.

#### Sand resistivity

**Figure 10:**
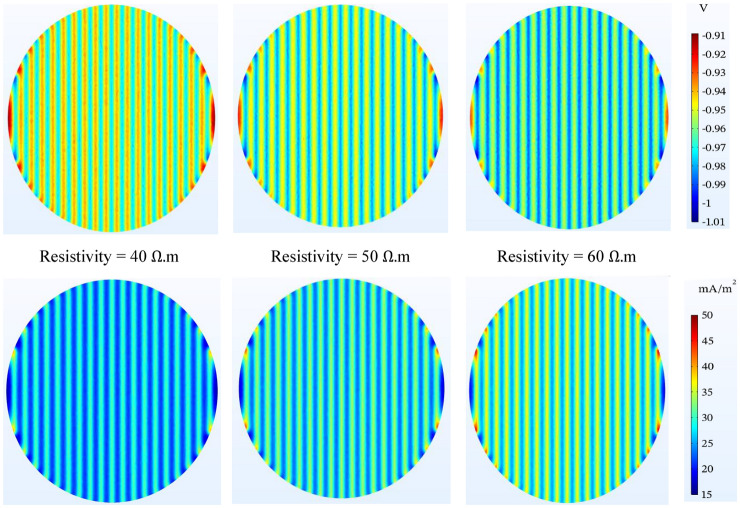
Potential ($\mathrm{V}-\mathrm{CSE}$) and current density ($\mathrm{mA}/{\mathrm{m}}^{2}$) distribution on the tank bottom at 0.5 m to the bottom plate and different resistivity of sand.

As seen in Fig. 10, the results of the simulation show that the effect of the sand resistivity is sensitive in the selected range, while with the increase of the sand resistivity, the anode resistance to electrolyte also increases (according to Rodenberg's relation), and for better protection of the tank bottom, more voltage will be needed, and by its nature, lower current density distribution will be required at a higher resistivity. An important point is that in Rodenberg's relation, with the increase of resistivity, the voltage value increases, but other parameters must also be considered.

#### Oxygen-limiting current density

**Figure 11:**
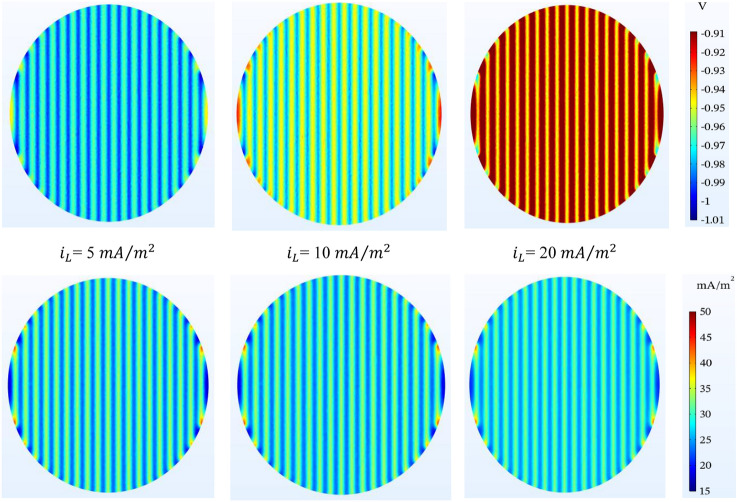
Potential ($V-CSE$) and current density ($\mathrm{mA}/{\mathrm{m}}^{2}$) distribution on the tank bottom at 0.5 m to the bottom plate and different oxygen-limiting current density.

For different values of oxygen-limiting current density, the conditions will be different. The higher the oxygen-limiting current density, the less protection the tank bottom will have. In Fig. 11, as can be seen in ${i}_{L}=20\mathrm{ mA}/{\mathrm{m}}^{2}$, the structure does not pass the protection criteria on the left and right edges while the protection criteria are established in other parts of the tank bottom. As it is known, the presence of oxygen will have a negative effect on cathodic protection. Also, in the current density distribution, this difference is sensible to some extent.

#### Anode depth

**Figure 12:**
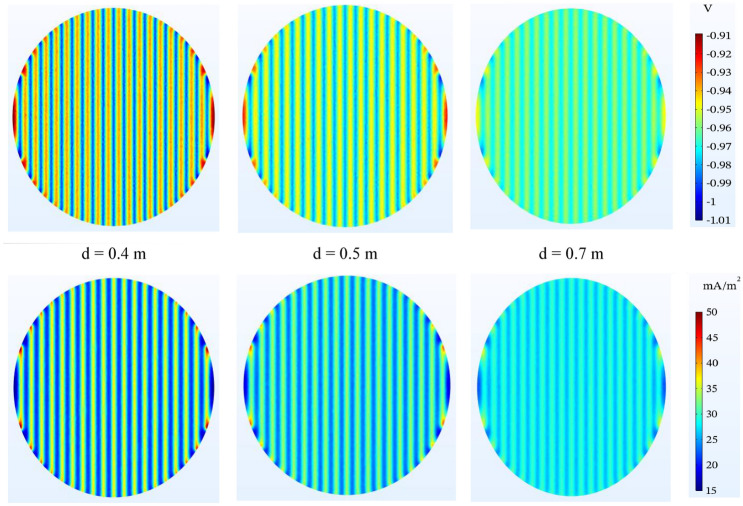
Potential ($V-CSE$) and current density ($\mathrm{mA}/{\mathrm{m}}^{2}$) distribution on the tank bottom at different distances to bottom plate and spacing by 1.5 m between ribbon anodes.

The burial depth of the anode has an effective effect on the tank bottom potential. The greater the burial depth, the more uniform the protection in the tank bottom and the smaller the difference between the maximum and minimum potential, while at a lower depth, this value will be greater. The current density distribution will be similar as shown in Fig. 12.

#### Anodic current

**Figure 13:**
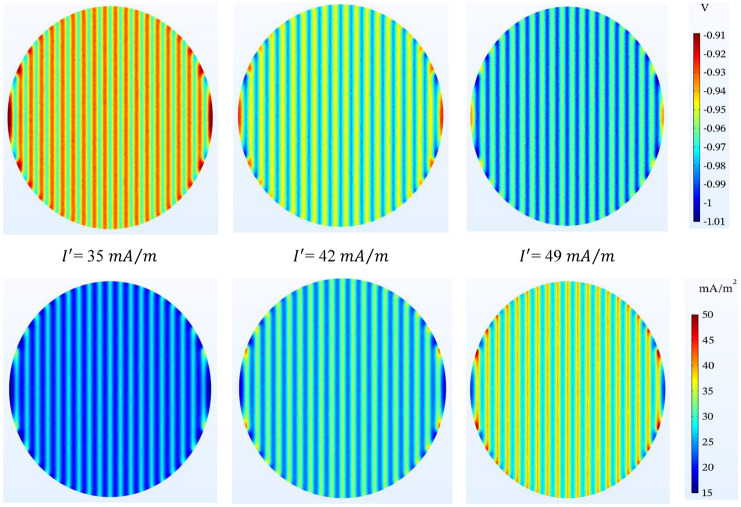
Potential ($V-CSE$) and current density ($\mathrm{mA}/{\mathrm{m}}^{2}$) distribution on the tank bottom at different anodic current and spacing by 1.5 m between ribbon anodes.

As expected, the simulation results in Fig. 13 show that as the amount of anodic current increases, the tank bottom is better protected. The numbers in the legend show that in all three cases the protection criterion is met, and the protection in 49 $\mathrm{mA}/\mathrm{m}$ is excellent at about 0.98 V. In the anodic current 35 $\mathrm{mA}/\mathrm{m}$, it can be seen that the protection potential in the mesh grid has values close to each other and around 0.93 V.

Table 9 is used for a better understanding of Figs. 8, 9, 10, 11, 12 and 13. In this table, the potential values in above the anode and mid-point are displayed for two status of simulation (Si) and Rodenberg equation (Ro). According to the NACE SP0169, results of simulation show that the potential values confirm the cathodic protection criteria in the range between − 850 and − 1150 mV with respect to ${\mathrm{Cu}/\mathrm{CuSO}}_{4}$ reference electrode except in some cases on the edges located in the shorter anodes (anodes placed in the left or right corners of the tank bottom.). Also, the greater difference in the simulation results and Rodenberg’s relation in ${V}_{r,MP}$ is because when the denominator of the fraction in the expression containing the natural logarithm function is reduced, this value has a significant change in the calculations and increases the difference greatly. As can be seen, the effect of oxygen-limiting current density, anodic current and soil resistivity shows the most fluctuations.

**Table 9:** Comparing the potential values in above the anode and mid-point of tank bottom potential by considering various parameters using ribbon anode mesh grid.

State	Soil resistivity ($\mathrm{\Omega m}$)	Anode spacing (m)	Anode depth (m)	Anodic current ($\mathrm{mA}/\mathrm{m}$)	Oxygen-limiting ($\mathrm{mA}/{\mathrm{m}}^{2}$)	${V}_{r,AA}$($-$ mV)		${V}_{r,MP}$($-$ mV)	
						Si	Ro	Si	Ro
(A) Changing the anode depth (m)									
1	50	1.5	**0.4**	42	10	**1000**	1080	**940**	890
2	50	1.5	**0.5**	42	10	**985**	990	**950**	870
3	50	1.5	**0.7**	42	10	**970**	880	**960**	820
(B) Altering the soil resistivity ($\mathrm{\Omega m}$)									
1	**40**	1.5	0.5	42	10	**980**	920	**945**	820
2	**50**	1.5	0.5	42	10	**985**	990	**950**	870
3	**60**	1.5	0.5	42	10	**1000**	1060	**955**	930
(C) Shifting the anode spacing (m)									
1	50	**1.4**	0.5	42	10	**985**	990	**955**	875
2	50	**1.5**	0.5	42	10	**985**	990	**950**	870
3	50	**1.6**	0.5	42	10	**980**	990	**940**	855
(D) Changing the oxygen-limiting current density ($\mathrm{mA}/{\mathrm{m}}^{2}$)									
1	50	1.5	0.5	42	**5**	**1000**	–	**965**	–
2	50	1.5	0.5	42	**10**	**985**	–	**950**	–
3	50	1.5	0.5	42	**20**	**940**	–	**875**	–
(E) Shifting the anodic operating current ($\mathrm{mA}/\mathrm{m}$)									
1	50	1.5	0.5	**35**	10	**965**	920	**920**	820
2	50	1.5	0.5	**42**	10	**985**	990	**950**	870
3	50	1.5	0.5	**49**	10	**1000**	1060	**960**	930

### Concentric ring system

To account for the small size of the wire anodes, we have utilized an extra fine element size during the meshing process. The resulting meshes include 247,069 mesh vertices, 1,419,510 tetrahedron elements, 56,235 triangle elements, 17,299 edge elements, and 107 vertex elements, with an average element quality of 0.8301. With the mesh size now established, we will proceed by generating and discussing the graph depicting the distribution of potential and current density changes for key parameters.

#### Anode depth

**Figure 14:**
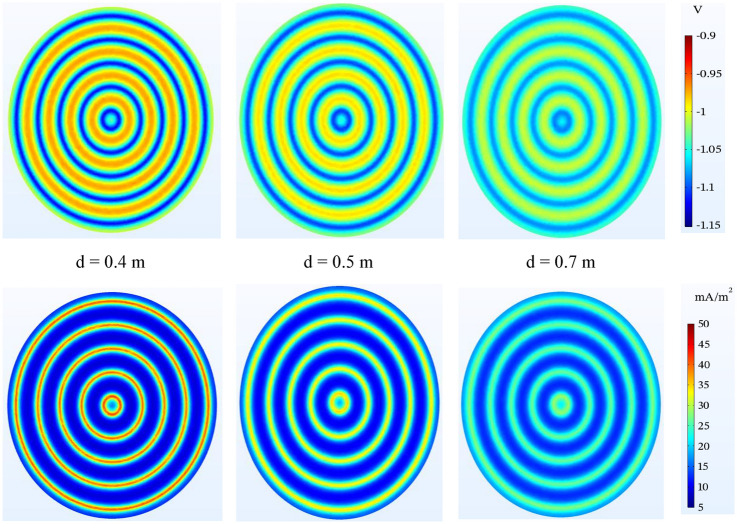
Potential ($V-CSE$) and current density ($\mathrm{mA}/{\mathrm{m}}^{2}$) distribution on the tank bottom at ${i}_{L}$= 5 $\mathrm{mA}/{\mathrm{m}}^{2}$ and different distance to bottom plate.

Anode burial depth is an effective factor in the quality of tank bottom potential. As seen in Fig. 14, increasing the burial depth of the anode makes the potential of most points of the tank bottom tend to lower values and the current is more evenly distributed. At a shallower depth, some points will receive more potential than the average value and others will receive less potential. The noteworthy point is the greater increase in the potential of this system compared to the mesh grid system.

#### Resistivity of sand soil

**Figure 15:**
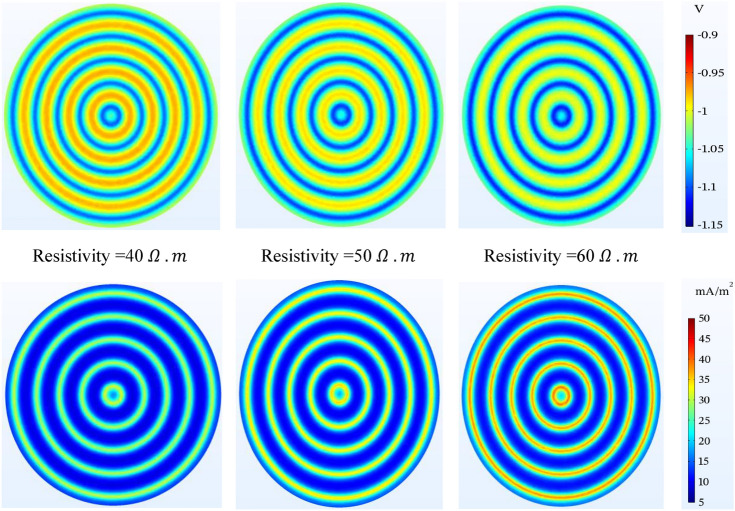
Potential ($V-CSE$) and current density ($\mathrm{mA}/{\mathrm{m}}^{2}$) distribution on the tank bottom at 0.5 m to bottom plate and different resistivity of sand.

In Fig. 15, similar to the simulation of the mesh grid system, in the concentric ring system, the potential changes due to the change of the sand resistivity in the selected range are small, with the difference that the potential change rate is in a better protection range and naturally, its effect will be greater in higher resistivity. For a better understanding of the changes, refer to Table 10.

**Table 10:** Comparing the potential values in above the anode and mid-point of tank bottom potential by considering various parameters using wire anode concentric rings.

State	Soil resistivity ($\mathrm{\Omega m}$)	Anode depth (m)	Anodic current ($\mathrm{mA}/\mathrm{m}$)	Oxygen-limiting ($\mathrm{mA}/{\mathrm{m}}^{2}$)	${V}_{r,AA}$($-$ mV)		${V}_{r,MP}$($-$ mV)	
					Si	Ro	Si	Ro
(A) Changing the anode depth (m.)								
1	50	**0.4**	60	5	**1130**	1290	**975**	840
2	50	**0.5**	60	5	**1110**	1170	**985**	830
3	50	**0.7**	60	5	**1085**	1020	**1000**	820
(B) Altering the soil resistivity ($\mathrm{\Omega m}$)								
1	**40**	0.5	60	5	**1100**	1050	**970**	780
2	**50**	0.5	60	5	**1110**	1170	**985**	830
3	**60**	0.5	60	5	**1120**	1300	**1000**	890
(C) Changing the oxygen-limiting current density ($\mathrm{mA}/{\mathrm{m}}^{2}$)								
1	50	0.5	60	**1**	**1115**	–	**1020**	–
2	50	0.5	60	**5**	**1110**	–	**985**	–
3	50	0.5	60	**10**	**1090**	–	**910**	–
(D) Shifting the anodic operating current ($\mathrm{mA}/\mathrm{m}$)								
1	50	0.5	**48**	5	**1090**	1050	**965**	780
2	50	0.5	**60**	5	**1110**	1170	**985**	830
3	50	0.5	**72**	5	**1130**	1300	**1010**	890

#### Oxygen-limiting current density

**Figure 16:**
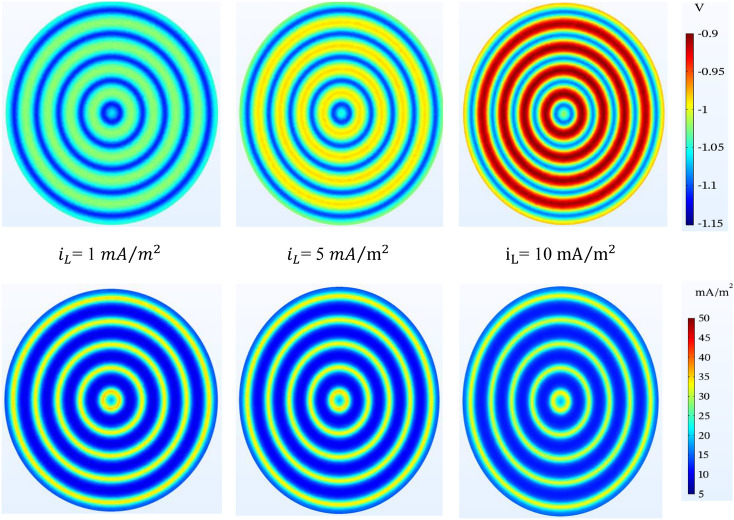
Potential ($V-CSE$) and current density ($\mathrm{mA}/{\mathrm{m}}^{2}$) distribution on the tank bottom at 0.5 m to bottom plate and different oxygen-limiting current density.

The presence of backfill around the wire anodes will have an effective role in the amount of oxygen available. The higher the oxygen-limiting current density, the less protection the tank bottom will have. With the presence of backfill, the amount of oxygen decreases, and cathodic protection is placed in a more favorable range. In Fig. 16, as seen in ${i}_{L}$= 10 $\mathrm{mA}/{\mathrm{m}}^{2}$, the structure experiences less protection compared to other current density values and the structure potential approaches the threshold of the protection criterion in some parts. So clearly the presence of oxygen will have a negative effect on cathodic protection. Also, this difference is moderate in the current density distribution.

#### Anodic current

**Figure 17:**
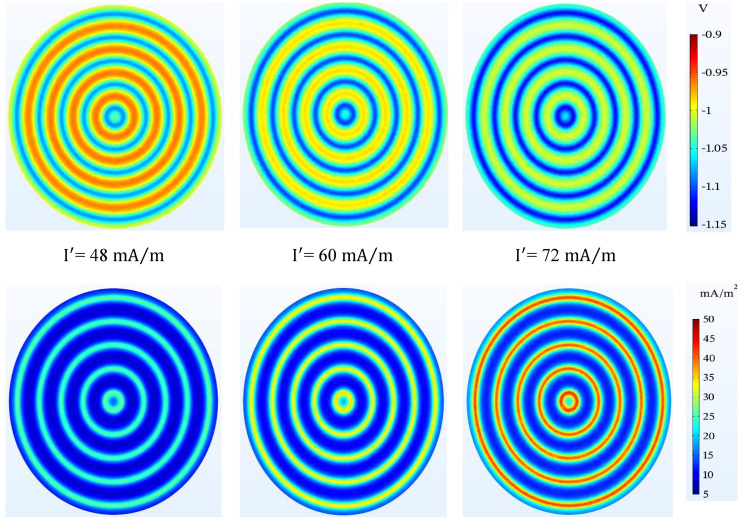
Potential ($V-CSE$) and current density ($\mathrm{mA}/{\mathrm{m}}^{2}$) distribution on the tank bottom at 0.5 m to bottom plate and different anodic current.

The effect of the amount of anodic current is noticeable in the level of protection of the tank bottom. The results of Fig. 17 show that with the increase of the anodic current, the potential of the tank bottom reaches a more favorable value and in all three cases the protection criterion is maintained. It is worth noting that the further increase of the passing current causes over-protection.

Table 10 is used for more effective presentation of Figs. 14, 15, 16 and 17. In this table, the potential values in above the anode and mid-point are displayed to compare 2 viewpoint of simulation (Si) and Rodenberg equation (Ro). Based on the NACE SP0169, results of simulation demonstrate that the potential values confirm the cathodic protection criteria. As can be seen, the effect of oxygen-limiting current density has been shown to be more significant than other parameters.

## Cost comparison of ribbon anodes in impressed current and sacrificial methods with wire anodes

Table 11 shows data about magnesium and zinc sacrificial ribbon anodes^40–42^. The purpose of this work is to compare the costs associated with sacrificial anodes and linear anodes (impressed current cathodic protection).

**Table 11:** Physical and electrochemical information about magnesium and zinc sacrificial ribbon anode.

Type	$\mathrm{Consumption rate}$(${\mathrm{C}}_{\mathrm{r}}$) ($\mathrm{kg}/\mathrm{A year}$)	$\mathrm{Electrochemical }capacity \left({C}_{a}\right)$ ($\mathrm{A h}/\mathrm{kg}$)	Ribbon dimensions (cm × cm)	Weight per meter ${\mathrm{m}}_{\mathrm{mg}/\mathrm{Zn}}$($(\mathrm{kg})$	Current (I) (A)	Efficiency (E) (%)
Mg	4	2200	0.94 × 1.9	0.36	17.6	50
Zn	10.71	820	2.5 × 3.2	3.57	17.6	90

In sacrificial anodes, after calculating the weight of the anode and then the required length, the cost of the used anodes is calculated. The procedure is presented below for Mg and Zn anode:

The weight of magnesium or zinc sacrificial anode is calculated over 40 years as follows:

29 $${\mathrm{m}}_{\mathrm{a}}=\mathrm{I }\cdot \mathrm{t}\cdot {\mathrm{C}}_{\mathrm{r}}/\mathrm{E}$$

where t (s) is time. The length of magnesium or zinc ribbon anode is given by the following equation:

30 $${\mathrm{L}}^{\prime}={\mathrm{m}}_{\mathrm{a}}/{\mathrm{m}}_{\mathrm{mg}/\mathrm{Zn}}$$

Also, the cost of magnesium or zinc ribbon anode is calculated:

31 $${\mathrm{C}}_{\mathrm{mg}/\mathrm{Zn},\mathrm{Total}}={\mathrm{L}}^{\prime} \times {\mathrm{C}}_{\mathrm{mg}/\mathrm{Zn}}$$

where ${\mathrm{C}}_{\mathrm{mg}/\mathrm{Zn}}$ is Cost per meter of Mg or Zn. Table 12 shows the results related to the comparison of the mentioned methods. As you can see, the cost of using sacrificial ribbon anodes is very high. Also the amount of sacrificial anodes required is a lot and this amount is technically unreasonable, and for these reasons, it is recommended to use MMO anodes in the cathodic protection of the tank bottom. In these calculations, the cost related to cables and drilling is not included.

**Table 12:** Economic comparison of different components of the protection system of ribbon and wire anodes^31,43^.

Type	Length of anodes (m)	Cost per meter/C ($)	Anode cost ($)	Rectifier cost ($)	Conductor bar cost ($)	Total cost ($)
Mg ribbon	15,645	4.5	4.5 × 15,645			70,400
Zn ribbon	2347	33	33 × 2347			77,500
MMO ribbon (C1)	446	13	13 × 446	8000	7 × 147	14,850
MMO ribbon (C2)	446	13	13 × 446	8000	7 × 117	14,650
MMO wire	230	30	30 × 230	8000		14,900

Design recommendations for linear anodes in cathodic protection systems should take into account the specific requirements of the tank and the surrounding environment. A combination of numerical analysis and practical considerations can be used to determine the most effective and cost-efficient system for each application. Table 13 provides a comparison of mesh grid system vs. concentric rings based on operational and economic analyses:

**Table 13:** Comparison of mesh grid system vs. concentric rings.

	Mesh grid system (C1)	Concentric rings
The need for field operations to install the anode	Yes (field-installed system)	No (assembled in the factory)
Need to weld (possibility of damage)	Yes (cutting and welding)	No
Oxygen generator as a depolarizer	Yes (without backfill)	Limited (with backfill)
Lifespan system (years)	20–30	30–60
Required length of anode (m)	446	230
Total cost ($)	14,850	14,900

Based on our study results and data, it can be concluded that the concentric rings method is more effective than the mesh grid system.

## Conclusions

Depending on the number of tanks, the diameter of the tanks, and the technology employed, different methods can be used to protect the bottom of the tanks. Nowadays, the use of linear MMO anodes such as wire and ribbon anodes is on the agenda, along with the use of geo membrane layer as a protective layer. The presence and implementation of the geo membrane layer plays an effective role in distributing the current on the bottom of the tank and preventing current leakage to the adjacent structures in the linear anodes. Here we summarize the key observations of our study:

1. Because the wire anodes along with backfill are assembled in the factory and installed on the site by marking, compared to the ribbon anodes that must be cut and welded on the site, installation is easier and more convenient.
2. Optimal spacing between conductor bars and a well-considered selection of the number of conductor bars adjacent to the feeding cables can effectively reduce the grid's internal resistance. This reduction is crucial for determining the appropriate sizing of the transformer rectifier. In our current study, the first configuration, which exhibits a more favorable selection, demonstrates an internal resistance value of 0.152 Ω, while the second configuration yields a higher value of 0.362 Ω.
3. The anode internal resistance of the concentric rings (backfilled wire anodes) is much less compared to the mesh grid system (ribbon anodes) because the dimensions of the wire anodes are in the order of cm and the dimensions of the ribbon anodes are in the order of mm and according to the equation $R=\rho l/A$; the smaller the area, the higher the internal resistance of linear anode.
4. Sand resistivity changes in the range of 40–60 $\mathrm{\Omega m}$ have sensible change in the cathodic protection potential of the tank bottom. As the soil resistivity ($\uprho$) increases, the resistance ($\mathrm{R}$) increases. Therefore, the increase in soil resistivity causes more potential shift and leads the potential distribution in a better protection range (according to Rodenberg’s relation). It should also be taken into account that increased resistance means less current is needed to achieve the required protection.
5. The spacing of anodes plays an effective role in better current distribution and choosing the optimal distance, taking into account the cost, and also confirming the protection criteria, is considered as one of the design requirements.
6. Oxygen content affects the required current of the structure for protection. Increasing the amount of oxygen causes a decrease in the level of protection and in some cases causes the protection criteria to be disapproved. In such a way, by reducing the oxygen-limiting current density from 10 to 1 $\mathrm{mA}/{\mathrm{m}}^{2}$, the potential values in mid-point, ${V}_{r,MP}$, reaches from − 0.91 to − 1.02 V and it shows that the reduction of oxygen-limiting current density has a significant effect on the protection of the structure.
7. Simulation results show that increasing the burial depth of the wire anode (0.4 $\stackrel{m}{\to }$ 0.7), causes a fine decrease in the potential values in above the anode, ${V}_{r,AA}$, ($-1.13V\stackrel{ }{\to }-1.085V$), and at the same time causes a more uniform distribution of the potential but this value should not be such that the achievement of the protection criterion is lost.
8. The simulation results show that the anodic operating current has an effective role in the protection potential. The higher level of anodic current, or in general, the higher rate of current produced by the anode, the tank bottom to be protected better and more uniformly. In the wire anode method, with the same conditions, with an increase of 25% of the anodic operating current, the average protection potential increases by 2% and improves the protection to the desired level. Also, a further increase in the anodic current does not indicate more favorable or much better conditions, and the optimal current should be estimated with a suitable pattern.
9. Among the ribbon anodes, MMO anodes have better performance than magnesium and zinc sacrificial anodes. The cost associated with the impressed current system method (MMO wire and ribbon anodes) is far lower than method of sacrificial ribbon anodes, and in the present study, this value for MMO anodes is approximately 20% of the magnesium and zinc ribbon sacrificial anode.
10. The length of the anode used in the cathodic protection of the tank bottom is different according to the method used. The length used in the MMO wire anode method is 230 m, which is 48.4%, 90.2%, and 98.53% shorter than MMO, zinc, and magnesium ribbon anodes, respectively.

## Future proposals

The following are suggested for future work:

1. The soil content should be chloride free and the amount of sulfide and sulfate should be as low as possible and it is recommended to be considered in future simulations.
2. The role of the geo membrane layer and its thickness should be investigated.
3. The presence of the conductor bars should be provided in the simulation.
4. The optimal position of the load conductor and power feed cable should be investigated.
5. The role of welding and its effect in the simulation should be considered by defining the resistance in several points and checking the amount of damage at those points and its effect on the required current density and potential.

### Supplementary Information

Supplementary Information.

## Data Availability

The datasets used and/or analyzed during the current study available from the corresponding author on reasonable request.
