# Supplementary material for: A comparative numerical evaluation of linear anode arrangements for enhancing above ground storage tank cathodic protection via mesh grid and concentric ring designs
Source: Sci Rep. 2023 Nov 10;13:19596. doi: 10.1038/s41598-023-44759-3 (PMC10638400; doi:10.1038/s41598-023-44759-3)
Supplement: Supplementary file 1 — Supplementary Information. [file 41598_2023_44759_MOESM1_ESM.docx]

**Supplementary material**

The amount of length needed to protect the tank bottom at spacing of 1.5 m with the knowledge of the grid radius and the distance between the anodes, is presented in detail below.

Table 0. Detail of Calculation for Spacing by *1.5 m* in ribbon anodes.

| *Mesh number* | *Grid*  *radius* | *Spacing bt anodes* | *Anode length (1^st^ quad)* | *Anode length (2^nd^ quad)* | *Anode length (3^rd^ quad)* | *Anode length (4^th^ quad)* |
| --- | --- | --- | --- | --- | --- | --- |
| *1* | *14.6* | *0* | *14.6* | *14.6* | *0* | *0* |
| *2* | *14.6* | *1.5* | *14.53* | *14.53* | *14.53* | *14.53* |
| *3* | *14.6* | *3* | *14.29* | *14.29* | *14.29* | *14.29* |
| *4* | *14.6* | *4.5* | *13.89* | *13.89* | *13.89* | *13.89* |
| *5* | *14.6* | *6* | *13.31* | *13.31* | *13.31* | *13.31* |
| *6* | *14.6* | *7.5* | *12.53* | *12.53* | *12.53* | *12.53* |
| *7* | *14.6* | *9* | *11.5* | *11.5* | *11.5* | *11.5* |
| *8* | *14.6* | *10.5* | *10.15* | *10.15* | *10.15* | *10.15* |
| *9* | *14.6* | *12* | *8.32* | *8.32* | *8.32* | *8.32* |
| *10* | *14.6* | *13.5* | *5.56* | *5.56* | *5.56* | *5.56* |
| *Ribbon mesh length in each quad* | | | *118.7* | *118.7* | *104.1* | *104.1* |
| *Total ribbon mesh length ( Spacing=1.5 m )* | | | | | | *445.6* |
